# Automated Solid-Phase Radiofluorination Using Polymer-Supported Phosphazenes

**DOI:** 10.3390/molecules180910531

**Published:** 2013-08-30

**Authors:** Bente Mathiessen, Fedor Zhuravlev

**Affiliations:** Hevesy Laboratory, Center for Nuclear Technologies, Technical University of Denmark, Risø Campus, Frederiksborgvej 399, P.O. Box 49, 4000 Roskilde, Denmark; E-Mail: beem@dtu.dk

**Keywords:** solid phase radiofluorination, dose-on-demand, polymer-supported phosphazene, [^18^F], multivariate analysis

## Abstract

The polymer supported phosphazene bases **PS**-P_2_^tBu^ and the novel **PS**-P_2_^PEG^ allowed for efficient extraction of [^18^F]F− from proton irradiated [^18^O]H_2_O and subsequent radiofluorination of a broad range of substrates directly on the resin. The highest radiochemical yields were obtained with aliphatic sulfonates (69%) and bromides (42%); the total radiosynthesis time was 35–45 min. The multivariate analysis showed that the radiochemical yields and purities were controlled by the resin load, reaction temperature, and column packing effects. The resins could be reused several times with the same or different substrates. The fully automated on-column radiofluorination methodology was applied to the radiosynthesis of the important PET radiotracers [^18^]FLT and [^18^F]FDG. The latter was produced with 40% yield on a 120 GBq scale and passed GMP-regulated quality control required for commercial production of [^18^F]FDG. The combination of compact form factor, simplicity of [^18^F]F− recovery and processing, and column reusability can make solid phase radiofluorination an attractive radiochemistry platform for the emerging dose-on-demand instruments for bedside production of PET radiotracers.

## 1. Introduction

Over the years positron emission tomography (PET) has developed into one of the most successful nuclear imaging modalities [1]. PET is a non-invasive, 10^−12^M/cm^3^-sensitive imaging method [2], able to provide quantitative biomolecular information regarding physiological processes in real time [3]. PET is currently finding applications in cardiology, neuroscience, oncology, gene therapy [4] and drug development [5]. Although more positron emitting radionuclides find their way into PET applications, fluorine-18 remains the most used PET radioisotope [6] due to its optimal half-life (*T_1/2_* = 109.8 min), high-yielding positron decay (97%, no γ-emission) and a low and narrow energy spectrum (E_avg_ = 249.3 keV, E_max_ = 633.5 keV), which translates into lower noise and higher image spatial resolution [7]. In a typical PET radiopharmacy setting, 200 GBq of cyclotron-produced [^18^F]F^−^ would be used to synthesize 15-20 doses of a given radiopharmaceutical and to ship it to a number of satellite PET centers. Due to high cost of establishing and maintaining the production facilities as well as difficulties associated with radiotracer development, the centralized PET radiopharmacies tend to have a lean and expensive radiotracer “menu”. This limits the diversity of existing, and creates a barrier to adoption of new PET radiotracers [8]. Allowing researchers and clinicians to produce a tracer of their choice wherever and whenever there is a need for it, is a new paradigm often referred to as the “dose-on-demand” radiotracer production. Whether the tracer is produced from a radioisotope made at a centralized facility, or made on-site using the emerging compact cyclotron/radiosynthesis instruments [9], the dose-on-demand approach requires a significant change in current radiosynthesizer technology. The preferred embodiment of the dose-on-demand concept is an affordably priced, GMP-compliant, fully automated, compact (preferably table-top) instrument featuring: (1) single dose on demand tracer delivery via tracer-specific kits; (2) ability to run custom radiosyntheses; (3) execution of back-to-back production runs with minimal cassette replacement and radiation exposure to the operator; (4) integrated quality control (QC) module; (5) easy maintenance and operational simplicity. While some of these challenges can be met with new engineering approaches [10], the development of simpler and more efficient radiochemistry remains among the top priorities in radiochemical research.

In radiofluorination, the bottleneck is often considered to be the process of [^18^F]F− recovery and activation. In routine production, the nanomolar amounts of cyclotron-produced [^18^F]F− are recovered from several milliliters of its water solution by absorption onto an anion-exchange resin followed by elution with aqueous acetonitrile/ K_2_CO_3_ and Kryptofix. The subsequent azeotropic evaporations yield the [^18^F]KF·Kryprofix complex in varying degrees of hydration and reactivity. The overall procedure requires multiple liquid and gas transfers resulting in lengthening of synthesis, diminished yields, and complex automation. Furthermore, the cartridge is not reusable and does not lend itself to efficient miniaturization. Significant efforts focused on eliminating the azeotropic evaporation step, for example, by using ionic liquids [11] or by eluting [^18^F]F^−^ with a strong organic base in the presence of protic additives [12]. In the light of potential applicability to dose-on-demand radiosynthesis, a more attractive approach would involve radiofluorination performed directly on the solid phase extraction (SPE) media. This would obviate the need for fluoride elution and azeotropic evaporation and allow for seamless integration of potentially reusable SPE resin into an automated dose-on-demand radiosynthesis module. The viability of radiofluorination at the point of fluoride capture was previously demonstrated [13,14], although the non-reusable aminopyridine-based resin suffered from poor stability and inconsistent [^18^F]F^−^ trapping and radiofluorination. Herein, we report fully automated on-column radiofluorination of a wide variety of substrates using reusable polymer supported phosphazenes.

## 2. Results and Discussion

**Scheme 1:**
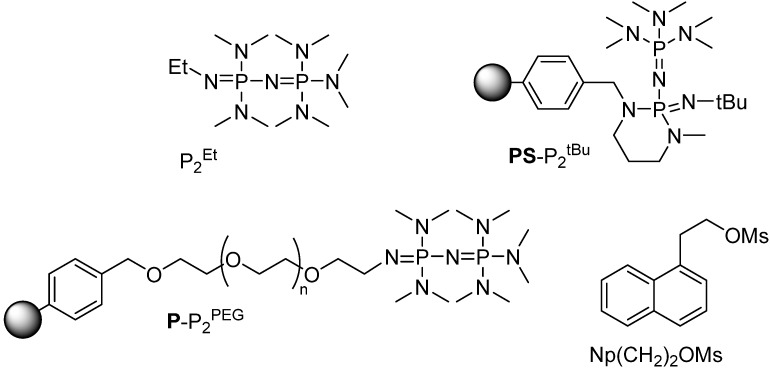
Some phosphazene bases and substrates used in this study.

**Scheme 2:**
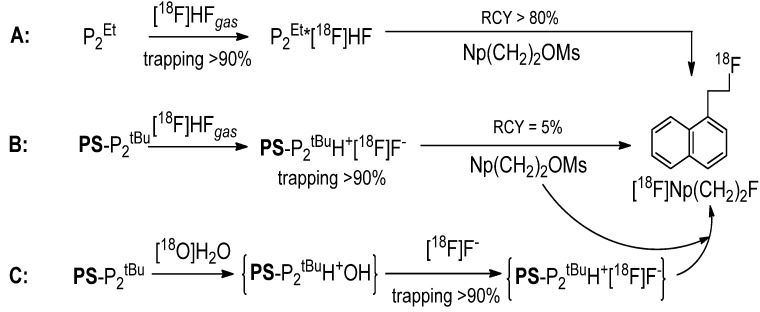
Radiofluorination using phosphazene bases.

We have recently reported that phosphazenium hydrofluoride [^18^F]P_2_^Et^·HF obtained by the reaction of P_2_^Et^ (Scheme 1) with [^18^F]HF [15] is an efficient radiofluorination reagent (Scheme 2A) [16].

To access the radiolabeling ability of its polymer-supported analogue, we reacted the commercially available polystyrene-supported **PS**-P_2_^tBu^ with [^18^F]HF(_gas)_ trapping 90% of activity as [^18^F]**PS**-P_2_^tBu^·HF. The subsequent reaction of the resin with the reference substrate Np(CH_2_)_2_OMs [16,17] (Scheme 1) yielded the desired [^18^F]Np(CH_2_)_2_F, albeit in only 5% radiochemical yield (RCY) (Scheme 2B).

RCY aside, we were encouraged by these results: based on Lemaire’s report, [12] one could expect the formation of [^18^F]**PS**-P_2_^tBu^H^+^F^−^ from the putative **PS**-P_2_^tBu^H^+^OH^−^ via a [^18^F]F^−^/OH^−^ anion exchange, whereas **PS**-P_2_^tBu^H^+^OH^−^ would result from the reaction of **PS**-P_2_^tBu^ with water (Scheme 2C). Therefore, we decided to treat the resin with aqueous [^18^F]F− and after drying, use the resulting [^18^F]F−-loaded resin for radiofluorination directly on the solid support. Indeed, this strategy proved fruitful, as **PS**-P_2_^tBu^ was able to extract the [^18^F]F− from the cyclotron-irradiated target water and deliver it to a variety of substrates (Table 1).

**Table 1:** Manual on-column radiofluorination using **PS**-P_2_^tBu^: Preliminary experiments.

Entry ^[a]^	Substrate	[^18^F]F^−^ trapping [%]	RCP [%] ^[b]^	RCY [%] ^[c]^
1	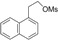	94	98	55
2	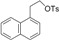	95	92	64
3	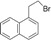	99	61	16
4	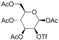	84	76	25
5	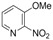	98	45	14

^[a]^ New column for each experiment; Substrate: 0.2 mmol (52µmol for entry 4); **PS**-P_2_^tBu^: 100 mg; [^18^F]F^−^: 200–800 MBq; Column drying: MeCN, 7 mL at 30 mL/h, 60 °C; Radiofluorination: toluene, 5 mL at 10 mL/h, 90 °C; ^[b]^ RCP: radiochemical purity; ^[c]^ RCY: decay-corrected radiochemical yield based on [^18^F]F^−^_(aq)_.

**Figure 1:**
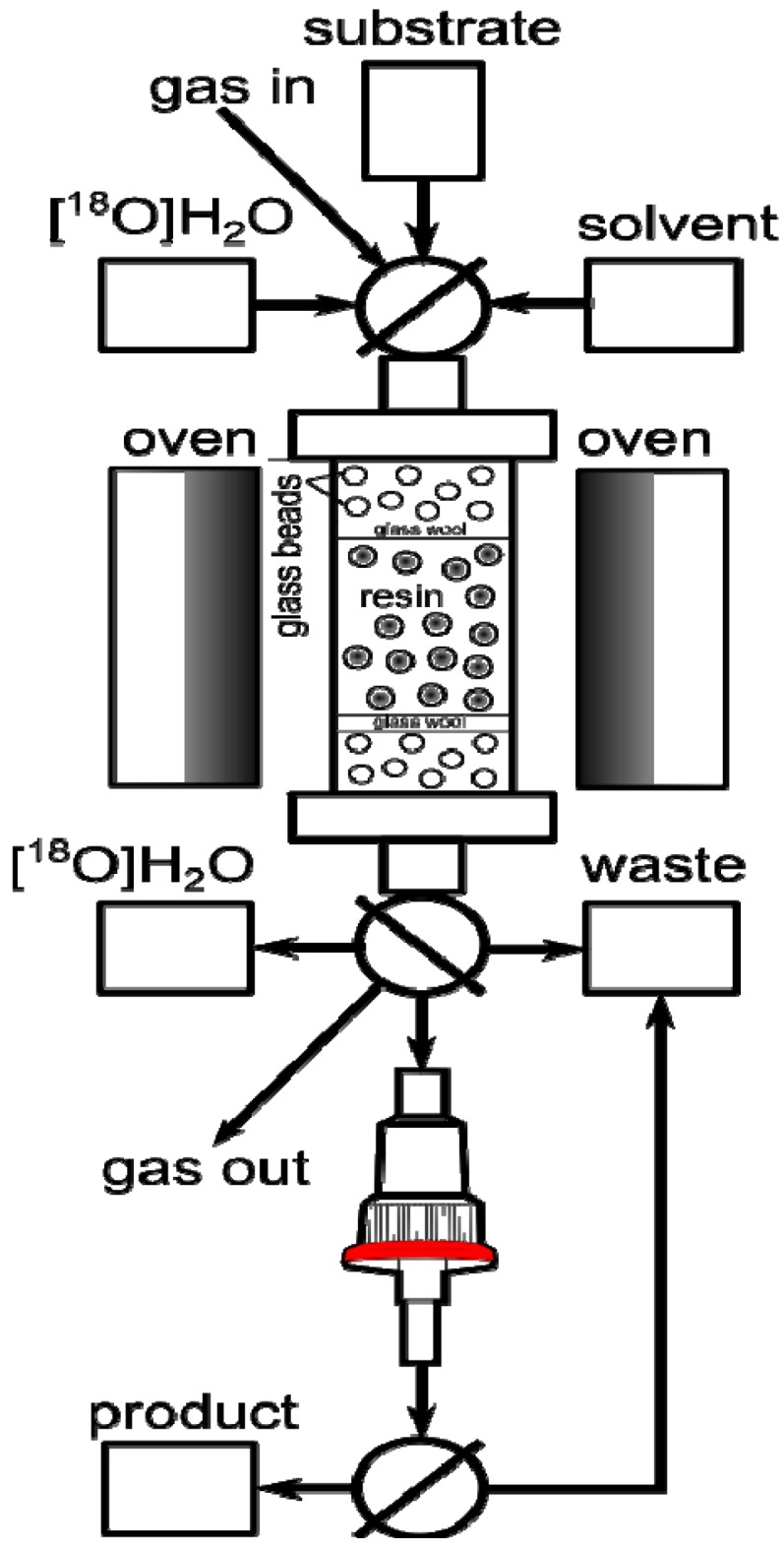
Automated radiosynthesis module for on-column radiofluorination.

The preliminary experiments listed in Table 1 were performed manually, by sequential single passes of the target water, acetonitrile and the solution of a substrate through a flexible PTFE tube packed with the resin and immersed in an oil bath. We were pleased to see that both the aliphatic (entries 1–4) and the aromatic (entry 5) substrates could be radiofluorinated. On the other hand, the fluoride trapping, RCP and RCY varied significantly across the substrates/runs. While some variabilities were undoubtedly substrate-related, others could be due to operational variations, warranting systematic screening and optimization studies performed with automated equipment. To that end we prepared a column consisting of a short glass tube fitted with standard SwageLok fittings and packed with **PS**-P_2_^tBu^ resin and glass beads to decrease back pressure. The column was connected to our home-made automated apparatus, where the necessary chemicals, solvents and gases could be remotely controlled and manipulated in a self-shielded and reproducible environment (Figure 1). Since the column could be as short as 5 cm, the size of the apparatus was limited only by the peripheral equipment, such as syringe pumps, valves and tubing.

In a typical automated experiment the target water was passed through the column, resulting in absorption of [^18^F]F− on the resin. The residual water was then removed from the column by rinsing of the column with dry MeCN. Further drying could be assisted by passing a flow of He through the column at 60 °C. Finally, the solution of substrate was passed through the column at a suitable flow rate and temperature, effecting on-column radiofluorination. If necessary, the unreacted [^18^F]F^−^ could be removed from the product using a silica Sep-Pak cartridge (Figure 1). At the outset we recognized that the screening and optimization studies would require the exploration of the experimental domain defined by various physical and chemical parameters. While changing one variable at a time is a common strategy, a more efficient approach is the use of multivariate experimental design known as the design of experiments (DoE) [18]. In DoE the experimental conditions (factors) are varied systematically and simultaneously according to a DoE algorithm and the outcome of the experiments (responses) are measured, thereby allowing for a statistically driven analysis of data in an optimal number of experiments. The final result is a model which describes the relationships between the factors and responses. The trapping efficiency of the resin was analysed first by varying the amount of resin (5–500 μmol), the volume of target water (0.5–3.5 mL), the flow rate (1.5–30 mL/min) and the number of times the column was used. The reaction matrix suggested by the DoE (Table 2) yielded an excellent single component partial least square (PLS) model (R^2^ = 0.91, Q^2^ = 0.89).

**Table 2:** On-column [^18^F]F− trapping using **PS**-P_2_^tBu^: trapping efficiency studies ^[a]^.

Number of times column was used ^[b]^	Resin [µmol]	Water [mL]	Flow rate [mL/min]	[^18^F]F− trapping [%]
1	5.8	0.5	1.5	77
4	5.8	0.5	1.5	67
1	509	0.5	1.5	100
4	509	0.5	1.5	100
1	5.5	3.5	1.5	57
4	5.5	3.5	1.5	58
1	499	3.5	1.5	100
4	499	3.5	1.5	100
1	5.1	30	30	56
4	5.1	30	30	52
1	501	30	30	100
4	501	30	30	99

^[a]^ Full factorial design at two levels reduced to 12 experiments and analyzed by PLS; ^[b]^ after each trapping the column was washed with dry MeCN (50 mL, flow rate 1.5 mL/min, rt) and dried with a flow of He (flow rate: 100 mL/min, 20 min).

**Figure 2:**
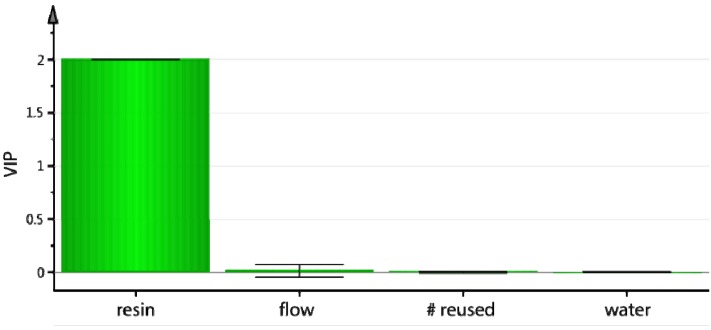
On-column [^18^F]F− trapping using **PS**-P_2_^tBu^: variables important for the projection (VIP) plot.

The variables important for the projection (VIP) plot indicated that the only factor controlling the trapping efficiency was the amount of resin (Figure 2). The column could be reused at least 3 times without any loss in [^18^F]F− trapping efficiency and without resin degradation.

**Figure 3:**
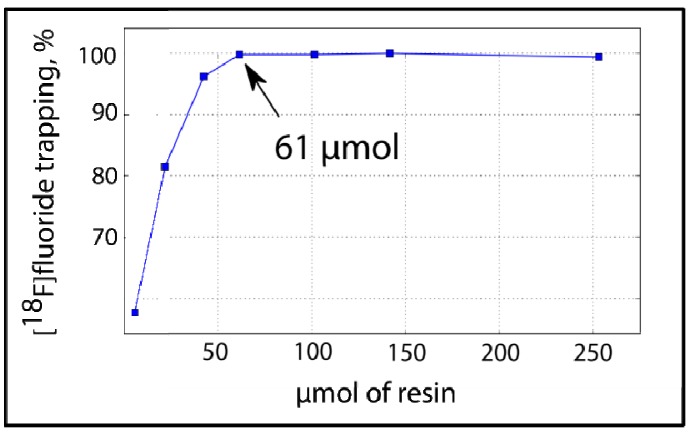
On-column [^18^F]F^−^ trapping using PS-P_2_^tBu^: Correlation between resin amount and [^18^F]fluoride trapping efficiency.

We found that the minimal amount of resin necessary to obtain 99% trapping efficiency was 61 μmol (Figure 3).

Although it is well known that hydration reduces fluoride nucleophilicity, some radiofluorination reactions show certain tolerance for water [11,12,16]. Since residual water entrapped in the resin could potentially influence both RCY and the radiochemical purity (RCP) we have attempted to optimize our drying protocol, varying the nature, the flow rate and the volume of the drying solvent, as well as the drying temperature (Table 3).

**Table 3:** Effect of column drying on radiofluorination of Np(CH_2_)_2_OMs with **PS**-P_2_^tBu^.

Entry ^[a]^	Drying solvent	Vol [mL]	Flow rate [mL/min]	Drying T [°C]	RCP [%] ^[b]^	RCY [%] ^[c]^
1	MeCN	1	1.5	25	88	32
2	Acetone	1	1.5	50	77	26
3	MeCN	10	1.5	50	77	18
4	Acetone	10	1.5	25	75	24
5	MeCN	1	20	50	74	20
6	Acetone	1	20	25	90	23
7	MeCN	10	20	25	85	32
8	Acetone	10	20	50	88	34
9	MeCN	5.5	10.75	38	85	25
10	MeCN	5.5	10.75	38	83	30
11	MeCN	5.5	10.75	38	91	31

^[a]^ New column for each experiment; **PS**-P_2_^tBu^: 180 µmol; [^18^F]F^−^: 100-1000 MBq; Radiofluorination: Np(CH_2_)_2_OMs: 52 µmol; toluene, 3 mL, flow rate 0.55 mL/min, 90 °C; ^[b]^ RCP: radiochemical purity; ^[c]^ RCY: decay-corrected radiochemical yield based on [^18^F]F^−^_(aq)_.

Despite good reproducibility (Table 3, entries 9–11), the analysis of the data showed little variability in RCY and did not result in a statistically valid model. No leaching of activity from the column was observed upon drying. For practical reasons, the drying protocol was adjusted to the use of MeCN (3–5 mL, 1.5 mL/min, 25 °C) for most of the following experiments.

The drying conditions established above were used to perform limited optimization studies where we looked at the influence of the amount of resin and the reaction temperature on RCP and RCY (Table 4). For consistency with our previous studies [16] the substrate loading was chosen at 52 μmol as the preliminary experiments showed that the high (150 μmol), medium (77.5 μmol) and low (5 μmol) substrate loading gave similar RCY.

Column packing effects, affecting the flow and likely to be responsible for some of the inter-column variability we have seen, were left out of optimization, as we did not find a reliable way to quantify them. It was also important to find out whether **PS**-P_2_^tBu^ resin could be reused not only at the trapping stage (Table 2), but also after radiofluorination. To that end, the trapping and radiofluorination sequence was repeated several times on the same column (columns A, C, D, and E, Table 4).

**Figure 4:**
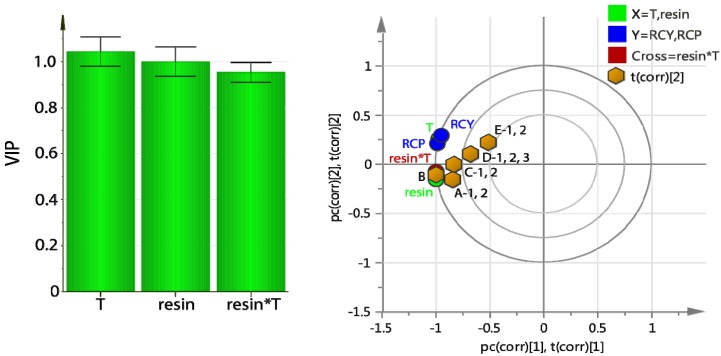
On-column radiofluorination of Np(CH_2_)_2_OMs using **PS**-P_2_^tBu^: variables important for the projection (VIP) plot (**left**) and bi-plot of loadings and scores (t), (**right**).

The optimization studies yielded a 2-component PLS model (R^2^ = 0.99. Q^2^ = 0.98) which indicated a positive correlation for both the amount of resin and the reaction temperature. Higher amount of resin and higher temperature (*T*) led to higher RCP and RCY (Figure 4, left). Interestingly, the cross term resin* T was also important and positively correlated. The high covariance between the X-values (*T, resin*) and the Y values (RCY, RCP) can be clearly seen in the bi-plot (Figure 4, right) as the factors (X) and responses (Y) are grouped together. The RCP and RCY for columns A and B, and to a lesser extent C, are dominated by the *resin* and resin* T terms. The *T* variable overlaps with RCP and RCY, suggesting that the three are highly correlated. The cross term can be interpreted as interaction: the effect of T on RCY and RCP depends on the amount of resin.

**Table 4:** On-column radiofluorination of Np(CH_2_)_2_OMs with PS-P_2_^tBu^: Optimization.

Entry ^[a]^	Column/run ^[b]^	PS-P_2_^tBu^, [μmol]	T [°C]	Trapping [%]	RCP [%] ^[c]^	RCY [%] ^[d]^
1	A/1	180	90	100	79	38
2	A/2	180	90	100	79	39
3	B	180	120	100	84	50
4	C/1	140	120	100	74	43
5	C/2	140	120	100	94	69
6	D/1	100	120	98	80	46
7	D/2	100	120	100	89	62
8	D/3	100	120	100	93	64
9	E/1	60	120	98	82	49
10	E/2	60	120	100	88	63

^[a]^ Trapping: [^18^F]F−/[^18^O]H_2_O, 200–800 MBq, 3mL, 1.5mL/min; Drying: MeCN, 3mL, 1.5mL/min; Radiofluorination: Np(CH_2_)_2_OMs: 52 μmol; toluene, 3 mL, 0.55 mL/min, 90 °C; ^[b]^ Letter-coded columns A–E differed in **PS-**P_2_^tBu^ loading and reaction temperature, the Arabic numerals refer to the number of consecutive trapping and radiofluorination runs performed on that particular column; ^[c]^ RCP: radiochemical purity; ^[d]^ RCY: decay-corrected radiochemical yield based on [^18^F]F^−^_(aq)_.

**Figure 5:**
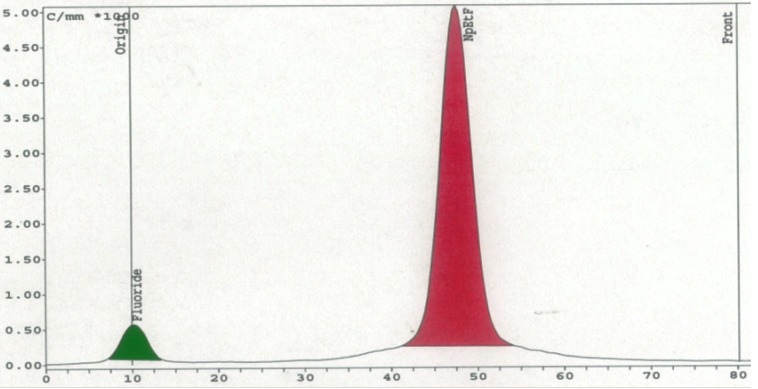
On-column radiofluorination of Np(CH_2_)_2_OMs with **PS**-P_2_^tBu^: Radio-TLC.

Importantly, Table 4 showed a consistent increase in the RCY and RCP when the columns were reused (columns A, C, D and E). The highest RCY and RCP (69% and 94%) were obtained on the second use of column C. Radio-TLC analysis showed that during radiosynthesis the only ^18^F-containing materials found in the reaction mixture eluted from the column were [^18^F]Np(CH_2_)_2_F and [^18^F]fluoride (Figure 5). The formation of the latter was consistent with the substrate-dependent elimination of [^18^F]HF triggered by the basicity of [^18^F] F−, as we have demonstrated for homogeneous fluorination with phosphazene hydrofluorides [16].

The remaining activity was retained on the column and could be removed neither with the fresh portion of substrate nor with a variety of solvents, such as toluene, DMF or water. Increasing substrate’s on-column residence time by recirculating the solution of Np(CH_2_)_2_OMs did not lead to any appreciable increase in RCY. The activity retained on the column after radiofluorination could indicate a physical entrapment of small fluoride anion within the polymer matrix, or a chemical deactivation due to reaction with the resin or impurities inside the resin.

**Scheme 3:**
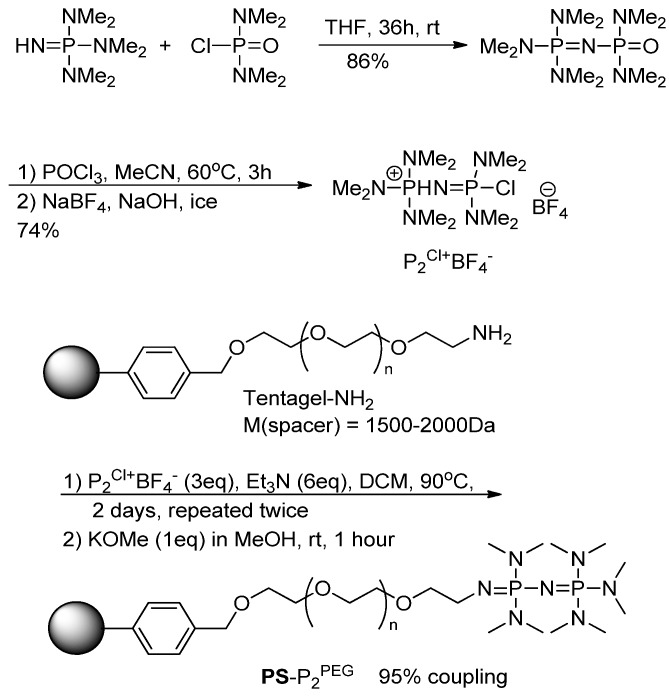
Synthesis of **PS**-P_2_^PEG^.

Next, we turned to investigation of polymer matrix effects. The mobility of polymer chains and hence the accessibility of functional groups depends on polymer swelling. The highly cross-linked **PS**-P_2_^tBu^ used in this study showed negligible swelling in MeCN, toluene and DMF. Therefore, swellable polymer matrix was sought after. The commercially available TentaGel-NH_2_ resin, which is based on PEG-grafted polystyrene matrix offers a high and largely solvent-independent swelling. From the design point of view the TentaGel-NH_2_ resin was also attractive as its alkyl-amino side chains could be easily functionalized with phosphazene residues. Consequently, the desired **PS**-P_2_^PEG^ was synthesized by applying the Schwesinger’s protocol [19] to the Tentagel –NH_2_ resin (Scheme 3). Both **PS**-P_2_^tBu^ and **PS**-P_2_^PEG^ could be stored at room temperature indefinitely.

The resulting **PS**-P_2_^PEG^ (95% phosphazene-functionalized) was reacted with Np(CH_2_)_2_OMs under the conditions optimized for **PS**-P_2_^tBu^. Unfortunately, due to its high swelling ability the microgel was prone to trapping air bubbles disrupting the uniform flow of the substrate through the column. Although this was partially rectified by careful degassing and recirculation of the starting material, the RCY topped at 38% (Table 5, entry 1). Similar to **PS**-P_2_^tBu^, the **PS**-P_2_^PEG^ resin could be reused: the sequential radiofluorination of Np(CH_2_)_2_OMs and Np(CH_2_)_2_OTs performed on the same column gave the desired [^18^F]Np(CH_2_)_2_F in 25% and 24% RCY correspondingly.

Table 5 summarizes the scope of the polymer supported bases used for radiofluorination of Np(CH_2_)_2_OMs. **PS**-P_2_^tBu^ gave the best RCY at 69% (entry 2) followed by **P**-P_2_^PEG^ at 38% (entry 1). Both resins trapped 99% of [^18^F]F−. The polymer-supported DIPEA (entry 3) and BEMP (entry 4) failed to trap fluoride, consistent with their lower pKa (11.4 and 27.6, correspondingly).

**Table 5:** Radiofluorination of Np(CH_2_)_2_OMs: the scope of the polymer supported bases.

Entry ^[a]^	Structure	[^18^F]F− trapping [%]	RCY [%] ^[b]^
1	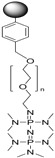	99	38
2	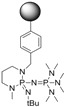	99	69
3	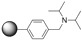	— ^[c]^	—
4	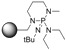	—	—

^[a]^ New column for each experiment; Trapping: [^18^F]F−/[^18^O]H_2_O, 500–1100 MBq, 3 mL, flow rate 1.5 mL/min; Drying: MeCN, 3 mL, flow rate 1.5 mL/min; Radiofluorination: Np(CH_2_)_2_OMs: 52 µmol; toluene, 3 mL, 0.55 mL/min, 90 °C; ^[b]^ RCY: decay-corrected radiochemical yield based on [^18^F]F^−^_(aq)_; ^[c]^ below detection limit.

The optimized conditions established for **PS**-P_2_^tBu^ and **PS**-P_2_^PEG^ were used to investigate the substrate scope. The results are presented in Table 6. The reactivity profile of both resins was similar to that observed for homogeneous radiofluorination with [^18^F]P_2_^Et^HF. [16] The sulfonates (Table 6, entries 1,2, and 7) were the best substrates for on-column radiofluorination with **PS**-P_2_^tBu^ and **PS**-P_2_^PEG^, delivering tetra-*O*-acetyl-[^18^F]FDG in 52% and 36% RCY, correspondingly (Table 5, entry 7). The halides could be radiofluorinated with **PS**-P_2_^tBu^, but not with **PS**-P_2_^PEG^ (Table 6, entries 3–5). The corresponding yields of FLT were only 7% and 5%, which is at the lower end of the currently reported homogeneous radiofluorination yields (8%–50%) [20]. The total radiosynthesis time for any substrate did not exceed 45 min.

**Table 6:** Radiofluorination with **P**-P_2_^tBu^ and **P**-P_2_^PEG^: The substrate scope.

Entry ^[a]^	Substrate	Trapping PS-P_2_^tBu^/PS-P_2_^PEG^ [%]	RCP ^[b]^ PS-P_2_^tBu^/PS-P_2_^PEG^ [%]	RCY ^[c]^ PS-P_2_^tBu^/PS-P_2_^PEG^ [%]
1	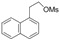	99/99	97/96	51/38
2	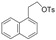	94/100	86/96	34/24
3	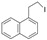	94/100	73/-	18/-
4	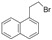	95/99	93/-	42/-
5	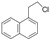	95/95	67/-	16/-
6	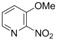	92/100	60/-	23/-
7	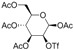	99/98	99/97	52/36
8	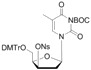	100/100	65/92	7/5

[a] New column for each experiment; Trapping: [^18^F]F−/[^18^O]H_2_O, 500–5000 MBq, 3 mL, flow rate 1.5 mL/min; Drying: MeCN, 3 mL, 1.5 mL/min; Radiofluorination: Substrate: 100 µmol; in toluene at 120 °C (aromatic substrates), tBuOH: MeCN 5:1 at 100 °C (FLT) or MeCN at 85 °C (FDG), flow 0.55 mL/min; Total radiosynthesis time between 35 and 45 min. [b] RCP: radiochemical purity; [c] RCY: radiochemical yield based on [^18^F]F^−^_(aq)_ as an average of two runs.

**Figure 6:**
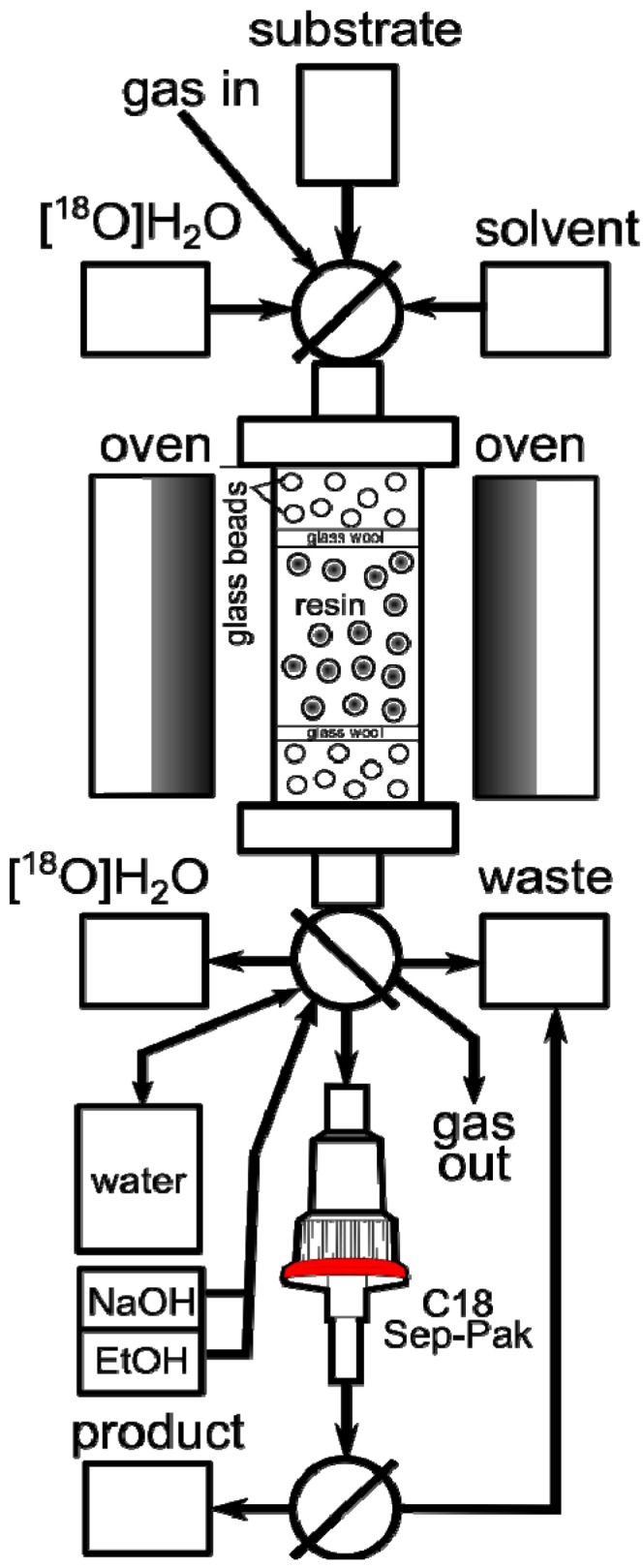
Automated radiosynthesis module for on-column radiosynthesis of [^18^F]FDG.

Finally, the on-column solid phase radiofluorination was applied to the automated radiosynthesis of [^18^F]FDG on a 120 GBq scale typically used for the commercial production of this radiotracer in our laboratory. The apparatus was adopted to accommodate the hydrolysis step (Figure 6).

Using **PS**-P_2_^tBu^ we achieved 98% of [^18^F]F− trapping efficiency obtaining the desired [^18^F]FDG in 40% RCY and 96.7% RCP as measured by the radio-HPLC and radio-TLC. The specific activity was measured to be higher than 11 GBq/μmol, the limit of detection of cold FDG by our HPLC. Importantly, the resulting formulation of on-column produced [^18^F]FDG met the stringent GMP quality control requirements used in our laboratory to release the commercial [^18^F]FDG produced by the conventional methodology (Table 7).

**Table 7:** Automated on-column radiosynthesis of [^18^F]FDG on 120 GBq scale: Quality Control.

Analysis	Result	Requirement (European Pharmacopoeia)
[^18^F]F−	0.5%	<5%
[^18^F]FDM	ND	<10%
[^18^F]FDG	97.8%	≥85%
Unhydrolyzed [^18^F]FDG	1.0%	<5%
RCP	96.7%	≥95%
[^19^F]FDG	<0.0167mg/mL	≤0.0167 mg/mL
Glucose	0.13mg/mL	No limit
Appearance	Clear, colorless	Clear, colorless or slightly yellow

## 3. Experimental

### 3.1. General

Unless otherwise noted, all synthetic steps were performed under an inert atmosphere of argon. Glassware and reaction vessels were dried in an oven at 160 °C overnight or flame-dried on the Schlenk line before use. All reagents were used as received without additional purification; toluene and THF were distilled from sodium benzophenone; other solvents were dried over activated molecular sieves. All radiochemical yields were decay-corrected. Polymer-supported P_2_^tBu^ (loading: 1.6 mmol/g), 2-tert-Butylimino-2-diethylamino-1,3-dimethylperhydro-1,3,2-diazaphosphorine (BEMP, loading: 2.3 mmol/g) and diisopropylaminoethyl (DIPEA, loading: 3 mmol/g) were obtained from Sigma Aldrich; the amine-functionalized polystyrene resin with polyethylene glycol spacer (TentaGel HL, particle size 160 µm, loading: 0.4 mmol/g, PEG: 1500–2000 Da) was purchased from Rapp Polymere GmbH. Mannose triflate was purchased from Sigma Aldrich and 3-*N*-Boc-5′-*O*-dimethoxytrityl-3′-*O*-nosyl-thymidine (FLT precursor) was purchased from ABX. The naphthalene halides or pseudohalides were purchased from Sigma Aldrich or synthesized as described elsewhere.

### 3.2. Instrumentation

^1^H, ^13^C and ^31^P-NMR spectra were recorded on a Bruker Avance II 500 instrument operating at 500, 126 and 202 MHz, respectively. ^19^F-NMR was recorded on a Brucer Avance DPX 250 instrument at 235 MHz. Mass spectrometry was performed on a Bruker Esquire 4000 ion-trap (IT) spectrometer equipped with electrospray ionization (ESI) interface. Thin-layer chromatography (TLC) was run on pre-coated plates of silica gel 60, F254 (Merck). Radio-HPLC was performed by using a Knauer HPLC System K501, equipped with a Knauer RI detector K2301 and CRA radioactivity detector 105 S-1 on a Carbopac PA10 4_25 mm Dionex column eluted with 0.1M NaOH at 1.0 mL min^−1^. Radio-TLC was performed with a Raytest MiniGita TLC scanner. The aqueous solutions of fluoride-18 were prepared by the ^18^O(p,n)^18^F reaction in a GE PETtrace cyclotron using a Ag or Nb target containing ~2.5 mL of 95%–98% enriched [^18^O]H_2_O irradiated by a 16.5 MeV proton beam at 55 µA. For automated radiosynthesis we used custom-made radiosynthesis robot controlled by LabView software.

### 3.3. Multivariate Analysis

Multivariate analysis was performed using MODDE 9.0 and SIMCA 13 software. The raw data were mean-centered and Pareto scaled; the Hotteling T2 values were below 95% for all models.

### 3.4. Synthesis of PS-P_2_^PEG^

TentaGel-NH_2_ was reacted with P_2_^Cl^*BF_4_ [19] by mixing the resin (200–1000 mg) with P_2_^Cl^*BF_4_ (3 eq. relative to the amine) and Et_3_N (dry, 9 eq relative to the amine) in DCM (dry, 5 mL) in a sealed glass ampule. The reaction mixture was then heated and shaken at 90 °C for three days. The procedure was repeated one more time in order to ensure better coupling. The resulting resin was deprotonated by reacting the resin with a mixture of KOMe (1 eq to amine) in MeOH (dry, 5 mL) for one hour giving the desired **PS**-P_2_^PEG^. The degree of resin functionalization by the phosphazene residues was estimated using [^19^F/^18^F]fluoride trapping in the following way: an aqueous solution of [^19^F]NaF (42.54 mg, 1.013 mmol) and [^18^F]F− (82.85 MBq) in 3.4 mL, corresponding to 0.012 mmol [^19^F]F^−^ MBq^−1^ was passed through a column containing **PS**-P_2_^PEG^ (204.58 mg, original amine content 0.4 mmol g^−1^) at 1 mL/min; the column was then washed with water (4 mL, flow 1 mL/min). The activity retained on the column was measured at 6.49 MBq [^18^F]F^−^, which corresponded to 0.078 mmol [^19^F]F^−^, and consequently, to 0.38 mmol of phosphazene residues per gram of resin (95% of the original amine functionalization).

### 3.5. [^18^F]Fluoride Trapping, RCP and RCY

[^18^F]fluoride trapping [%] was based on target water and calculated as: Trapping = 100%* (Activity ^added to the column^ – Activity ^eluted from the column^)/Activity ^added to the column^. The RCP was determined by radio-TLC or radio-HPLC and calculated as: RCP = (Area^product^/Total Area)*100%. The RCY was determined by the dose calibrator and calculated as RCY=Activity^product^*RCP/Activity[^18^F]^F− added to the column^.

### 3.6. Manual Radiofluorination of [^18^F]Np(CH_2_)_2_OMs with [^18^F]HF and PS-P_2_^tBu^ (Scheme 2, B)

A PTFE tube (OD = 0.64 cm) was packed with **PS**-P_2_^tBu^ (100–200 mg) and 1200 mg of glass beads and connected to a PE vial containing 4 mL of 98% H_2_SO_4_. The PE vial was connected to an Ar inlet and Ar was bubbled through sulphuric acid at a rate of 150–300 scc/min. [^18^F]F^−^_(aq)_ (1 mL, 200–500 MBq) was added and the vial was heated for 30 min at 80 °C in an ultrasound bath while being irradiated at 35 kHz under the flow of Ar. The resulting [^18^F]HF was carried by argon flow to the column. After 30 min the column was placed in an oil bath at 100 °C and a solution of NpEtOMs (200 µmol) in toluene (dry, 5 mL) was passed through the column using a syringe pump (flow rate 10 mL/h). Toluene (dry, 5 mL) was passed through the column to elute the residual product. The fluorinated product was analyzed by radio-TLC (eluent heptane:EtOAc 80:20). Trapping = 95%, RCP = 73%, RCY = 5%. Total time of radiosynthesis was up to 90 min.

### 3.7. General Procedure for Manual Solid-Phase Radiofluorination (Scheme 2 C and Table 1)

An aliquot of [^18^F]F^−^_(aq)_ (1.0 ml, 200–800 MBq) was mixed with water (3 mL) and passed through the column (PTFE tube, **PS**-P_2_^tBu^: 100 mg; glass beads: 1200 mg). MeCN (dry, 7 mL) was then passed through the column (flow rate: 30 mL/h, duration 10 min, rt to 60 °C) followed by Ar until excess of solvent was removed. A substrate (52–200 µmol) was dissolved in toluene (dry, 5 mL) and the solution was passed through the column (flow rate 10 mL/h, duration 30 min) while heating the column at 90 °C. Thereafter, toluene (dry, 5 mL) was passed through the column (flow rate 20 mL/h, duration 15 min) at 90 °C to elute the remaining product. The fluorinated products were analyzed by radio-TLC (eluent heptane:EtOAc 80:20 for the naphthalene derivatives, MeCN:H_2_O 95:5 for Ac_4_-FDG, DCM:MeOH 9:1 for hydrolyzed FLT, petroleum ether:EtOAc 3:1 for the pyridine derivative) Total time of radiosynthesis was 60–90 min.

### 3.8. General Procedure for Automated Solid-Phase Radiofluorination Using PS-P_2_^tBu^

An aqueous solution of [^18^F]F− (0.5–3.5 mL, flow rate 0.5–30 mL/min) was passed through the column (borosilicate glass tubing, ID 4 mm, OD 0.6 cm, length 12 cm) packed with the resin (5–500 µmol; glass beads to fill the remaining volume). To remove the bulk of water, the column was flushed with He_(gas)_ (flow rate 100 mL/min, 3 min). The column was dried by passing MeCN or acetone (1–10 mL, flow rate 1.5–20 mL/min) through, followed by a helium flush (100 mL/min, 2–5 min). The column was primed by passing the radiofluorination solvent (MeCN for mannose triflate, MeCN/tBuOH 1:5 for FLT-ONs, toluene for the naphthalene derivatives and 2-nitro-3-methoxypyridine, dry, 4 mL, flow rate 2 mL/min) through the column at room temperature followed by the substrate (50–100 µmol) dissolved in the radiofluorination solvent (dry, 3 mL) at 60–120 °C (flow rate 0.55 mL/min). The radiofluorination solvent (dry, 2 mL, flow rate 0.55 mL/min) was then passed through the column again to elute the remaining product. The reaction mixture was analyzed by radio-TLC (eluent heptane:EtOAc 80:20 for the naphthalene derivatives, MeCN:H_2_O 95:5 for FDG, DCM:MeOH 9:1 for hydrolyzed FLT, petroleum ether:EtOAc 3:1 for the pyridine derivative. Total time of radiosynthesis was 35–45 min.

### 3.9. General Procedure for Automated Solid-Phase Radiofluorination Using PS-P_2_^PEG^

The apparatus and column was degassed before use by passing through MeCN (5 mL, 1.5 mL/h) followed by water (5 mL, 1.5 mL/h). An aqueous solution of [^18^F]F^−^ (3.5 mL, flow rate 1.5 mL/min) was passed through the column (borosilicate glass tubing, I.D. 4 mm, OD 0.6 cm, length 12 cm) packed with the resin (200–500 µmol). Residual water was removed by passing through MeCN (5 mL, flow rate 1.5 mL/min). The column was primed by passing the radiofluorination solvent (MeCN for mannose triflate, MeCN/tBuOH 1:5 for FLT-ONs, toluene for the naphthalene derivatives and 2-nitro-3-methoxypyridine, dry, 4 mL, flow rate 1.5 mL/min) through the column at room temperature followed by the substrate (50-100 µmol) dissolved in the radiofluorination solvent (dry, 3 mL) at 60–120 °C (flow rate 0.55 mL/min). The radiofluorination solvent (dry, 2 mL, flow 0.55 mL/min) was then passed through the column again to elute the remaining product. The reaction mixture was analyzed by radio-TLC (eluent heptane:EtOAc 80:20 for the naphthalene derivatives, MeCN:H_2_O 95:5 for FDG, DCM:MeOH 9:1 for hydrolyzed FLT, petroleum ether:EtOAc 3:1 for the pyridine derivative. Total time of radiosynthesis was up to 45 min.

### 3.10. Washing Procedure for Back-to-Back Radiofluorination

After each trapping-radiofluorination sequence the column was washed with dry MeCN (50 mL, flow rate 1.5 mL/min, rt) and dried with a flow of He (flow rate: 100 mL/min, 20 min).

### 3.11. Large Scale Automated [^18^F]FDG Synthesis using P-P_2_^tBu^ Column

Mannose triflate together with the solvents and reagents used for hydrolysis of the product was acquired from ABX Advanced Biochemical Compounds (Radeberg, Germany) and are of pharmaceutical grade quality manufactured according to GMP standards. The cyclotron irradiated target water containing [^18^F]F− (3.0 mL, flow rate 1.5 mL/min, 119 GBq) was passed through the column (borosilicate glass, **PS**-P_2_^tBu^: 100 µmol; glass beads to fill the remaining volume) followed by He_(gas)_ (flow rate 100 mL/min, 2 min). MeCN (dry, 5 mL, flow 1.5 mL/min) was passed through the column followed by He_(gas)_ (100 mL/min, 3 min) in order to dry the column completely (trapped 117 GBq of [^18^F]F^−^, 98% trapping efficiency). MeCN (dry, 4 mL, flow 2 mL/min) was passed through the column at room temperature followed by mannose triflate (25 mg, 52 µmol) dissolved in MeCN (dry, 3 mL) at 85 °C (flow 0.55 mL/min). MeCN (dry, 2 mL, flow 0.55 mL/min) was then passed through the column to elute the remaining product. The two fractions were mixed with water (total volume 50 mL) and passed through a tC C-18 Sep Pak cartridge (preconditioned using 5.0 mL EtOH and 5.0 mL water) to trap the unhydrolyzed [^18^F]FDG. The cartridge was washed with water (5 mL). NaOH (2.0 M, 0.5 mL) was added to the cartridge in order to hydrolyze the compound (duration 5 min) and the hydrolyzed product was eluted with water (5 mL) to a product vial containing saline (9 mg/mL NaCl, 0.64% EtOH, total volume 14 mL) and a buffer solution (citrate buffer, 6 mL, pH 1.3). Full QC analysis confirmed that the product was [^18^F]FDG (49 GBq, RCP 97%, RCY 41%).

## 4. Conclusions

In conclusion, we have demonstrated that polymer supported phosphazenes **PS**-P_2_^tBu^ and the novel **PS**-P_2_^PEG^ allowed for efficient extraction of [^18^F]F− from the target water and subsequent radiofluorination of a broad range of substrates directly on the resin, thus simplifying radiosynthesis and QC by avoiding [^18^F]F− elution, azeotropic evaporation and kryptand addition. The highest radiochemical yields were obtained with aliphatic sulfonates (69%) and bromides (42%); the total radiosynthesis time for any substrate did not exceed 45 min. We found that the RCY and RCP were controlled by the amount of resin, reaction temperature, and column packing effects. The same resin could be used at least 3 times with the same substrate and at least two times with two different substrates. The incorporation of the compact phosphazene resin column into a remotely controlled programmable dispensing system resulted in a solid phase radiosynthesis module which was used for automated radiosyntheses, including FLT and a large scale production of [^18^F]FDG. Although at this stage of development the solid phase RCY are generally ~10%–30% behind those obtained homogeneously, the large scale production of GMP-quality [^18^F]FDG with 40% RCY and 97% RCP clearly demonstrates the potential of solid phase radiofluorination methodology. We believe that the combination of compact form factor, streamlined [^18^F]F− recovery and processing, and column reusability will make the solid phase radiofluorination an attractive radiochemistry platform for the emerging dose-on-demand instruments for bedside production of PET radiotracers.

## Supplementary Materials

Supplementary materials can be accessed at: http://www.mdpi.com/1420-3049/18/9/10531/s1.
