# Supplementary material for: Automated Solid-Phase Radiofluorination Using Polymer-Supported Phosphazenes
Source: Molecules. 2013 Aug 30;18(9):10531–47. doi: 10.3390/molecules180910531 (PMC6270316; doi:10.3390/molecules180910531)
Supplement: Supplementary file 1 [file molecules-18-10531-s001.pdf]

Supplementary Materials

Schwezinger Precursor for P-P_2^{PEG} Synthesis

Bis(dimethylamino){[tris(dimethylamino)phosphoranylidene]-amino}phosphorane Oxide (P_2 oxide)

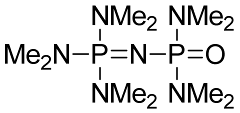

Imino-tris(dimethylamino)phosphorane (2440 µL, 13.40 mmol) was added to a 25 mL roundbottom flask [flame dried, backfilled with Ar(g)] followed by THF (dry, 4.0 mL). The solution was cooled to 0 °C and N,N,N',N'-tetramethylphosphorodiamidic chloride (1000 µL, 6.70 mmol) was added slowly by syringe pump (flow 2 mL/h). The reaction mixture (yellow with white precipitate) was then stirred at room temperature for 36 h under slight vacuum. Imino-tris(dimethylamino)phosphonium hydrochloride was removed by filtration, the solvent removed by rotevap and the resulting oil (yellow with trace amount of a crystalline compound) was dried under high vacuum. This crude product was destilled by a Kugelrohr apparatus 180 °C/0.2 Torr. Yield 1159 mg (55%) as colorless oil. ^{1}H-NMR (500 MHz, $CDCl_3$, 25 °C, TMS): δ = 2.65 (d, J = 10 Hz, 12H), 2,69 (d, J = 10 Hz, 18H); ^{13}C-NMR (126 MHz, $CDCl_3$, 25 °C, TMS): δ = 37.16 (d, J = 3.8Hz), 37.68 (d, J =2.5 Hz); ^{31}P-NMR (202 MHz, $CDCl_3$, 25 °C, TMS): δ = 11.91 (d, J = 48 Hz), 21.0 (d, J = 46 Hz).

1-chloro-1,1,3,3,3-pentakis(dimethylamino)-1λ5-diphosphazen-3-iumtetrafluorborate (P_2^{Cl}*BF_4)

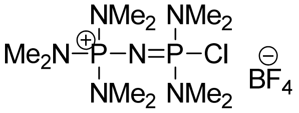

P_2 oxide (1159mg, 3.71 mmol) and MeCN (dry, 3.5 mL) was added to a 10mL round bottom flask [flame dried, backfilled with Ar(g)]. $POCl_3$ (3.71 mmol, 350 µL) was added slowly and the reaction mixture was stirred at 60 °C for 4 h. After cooling the solvent was removed by rotevap and the resulting oily residue was dissolved in DCM (4 mL). The solution was added to a vigorously stirred mixture of $NaBH_4$ (3.75 mmol, 419.29 mg) and NaOH (7.42 mmol, 298.33 mg) in ice (6g). As the ice melted the mixture turned very thick, but after further stirring this dissolved and a two-phase system was formed. The organic phase was concentrated by rotevap and dried in high vacuum at 50 °C. The resulting residue was dissolved in EtOAc/PrOAc (3:1, enough to reach a clear solution, about 30 mL). The solution was cooled to −50 °C for crystallization. The mother liquor was removed and the crystals washed with precooled PrOAc (10mL) and dried under high vacuum. Yield 1141mg (74%) as colorless deliquescent crystals.^{1}H-NMR (500 MHz, $CDCl_3$, 25 °C, TMS): δ = 2.74 (d, J = 10 Hz, 18H), 2.84 (d, J = 15 Hz, 12H); ^{13}C-NMR (126 MHz, $CDCl_3$, 25 °C, TMS): δ = 36.90 (d, J = 3.8Hz), 37.21 (d, J = 3.8 Hz); ^{31}P-NMR (202 MHz, $CDCl_3$, 25 °C, TMS): δ = 16.21 (d, J = 57 Hz), 23.07 (d, J = 57 Hz); ^{19}F-NMR (235 MHz, $CDCl_3$, 25 °C, TMS): δ = −159 (s with two shoulders).

Radio TLC Traces

Table S1. R_f values and TLC eluents for the radiofluorinated products.

Substrate	Product	R_f	Solvent
Naphtalene analogues	[^{18}F]Np(CH_2)$_2$F	0.50 ± 0.10 *	Heptane/EtOAc 4:1
Mannose triflate	[^{18}F]FDG	0.45±±0.05	MeCN:H_2O 95:5
3-Methoxy-2-nitropyridine	[^{18}F]3-Methoxy-2-fluoropyridine	0.26	Petroleum ether:EtOAc 3:1
FLT precursor	Unhydrolyzed [^{18}F]FLT	0.84	EtOAc:EtOH 1:1

* Always in correspondence with co-spotted reference compound [^{19}F] Np(CH_2)$_2$.

TLC trace [^{18}F]Np(CH_2)$_2$F

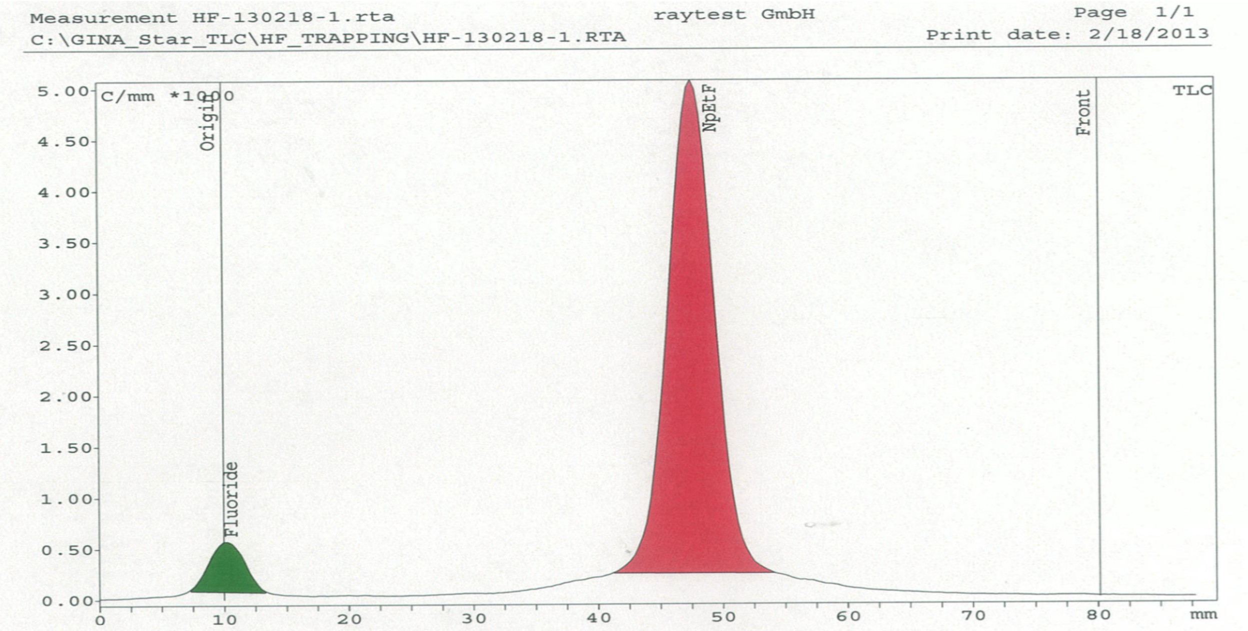

Integration TLC

Substance	R/F	%Total %	Type	Area Counts	%Area %
Fluoride	0.005	5.42	BB	1641.29	7.29
NpEtF	0.536	68.85	BB	20865.79	92.71
Sum in ROI				22507.07	
Total area				30306.00	
Area RF				29289.00	
Ext. BKG				0.00 C/mm	

TLC trace [18F]3-Methoxy-2-fluoropyridine

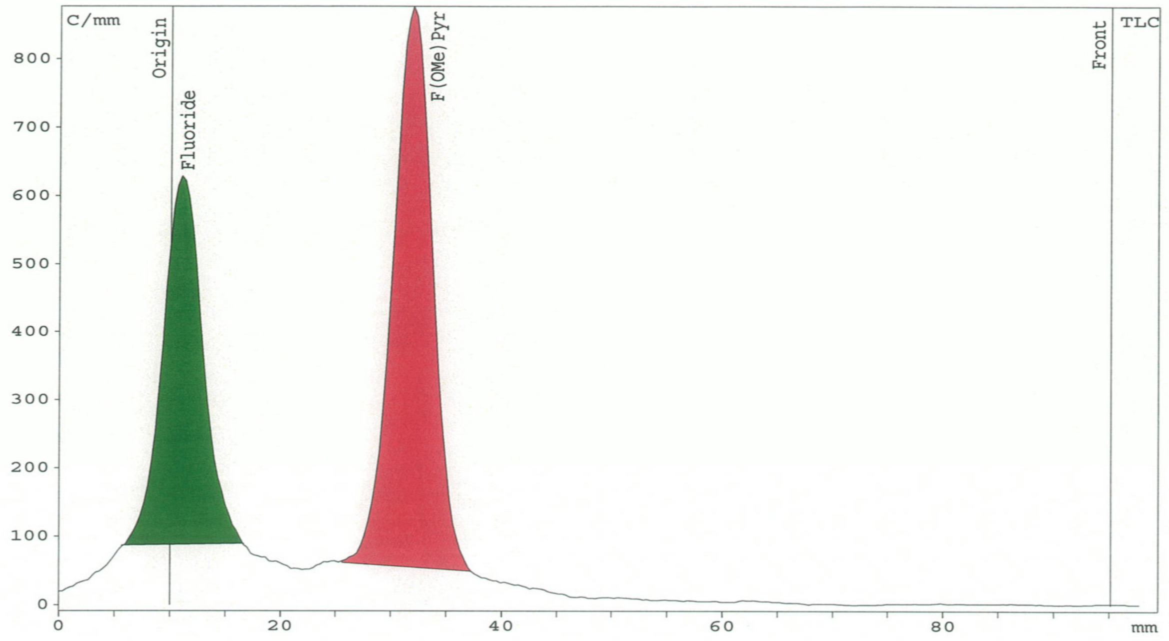

Integration TLC

Substance	R/F	%Total %	Type	Area Counts	%Area %
Fluoride	0.012	26.52	BB	2274.857	39.88
F(OMe)Pyr	0.258	39.98	BB	3429.500	60.12
Sum in ROI				5704.357	
Total area				8577.000	
Area RF				7570.000	
Ext. BKG				0.00 C/mm	

TLC trace unhydrolyzed [18F]FLT

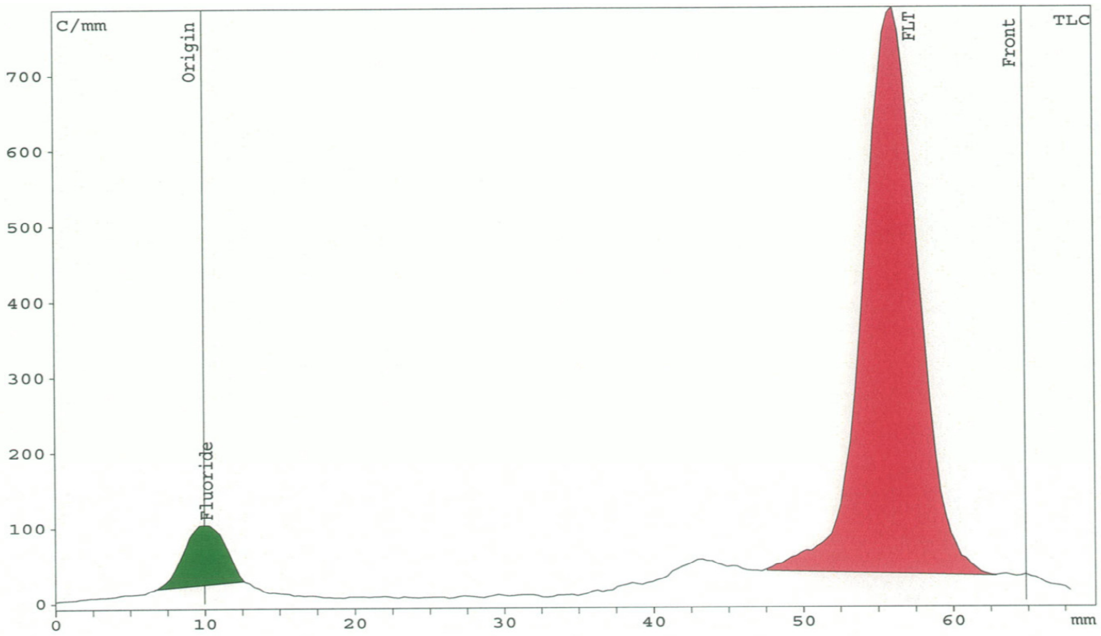

Integration TLC

Substance	R/F	%Total %	Type	Area Counts	%Area %
Fluoride	0.000	5.19	BB	260.000	7.55
FLT	0.842	63.58	BB	3184.571	92.45
Sum in ROI				3444.571	
Total area				5009.000	
Area RF				4684.000	
Ext. BKG				0.00 C/mm	

It was assumed that the minor impurity visible at about Rf 0.7 is labeling of the nosyl (4-nitrobenzylsulfonyl) group by substitution of the nitro group. Further studies were not carried out.

HPLC and TLC of Big Scale [^{18}F]FDG

Table S2. Retention times and areas for HPLC RI and radiodetector trace of [^{18}F]FDG.

	Ret. time	Area %	Figure
[^{18}F]F$^-$	5.33	0.52	1 + zoom
[^{18}F]FDG	9.23	97.76	1 + zoom
[^{18}F]FDM *	Not detected	Not detected	1 + zoom
[^{19}F]FDG	Not detected	Not detected	2

* Ret time ([^{19}F]FDM) = 8.2 established by HPLC analysis of reference compounds.

HPLC was performed using a Knauer HPLC System K501, equipped with a Knauer RI detector K2301 and CRA radioactivity detector 105 S-1 on a Carbopac PA10 4_25 mm Dionex column eluted with 0.1M NaOH at 1.0 mLmin^{-1}.

Figure S1. Radiodetector trace of [^{18}F]FDG.

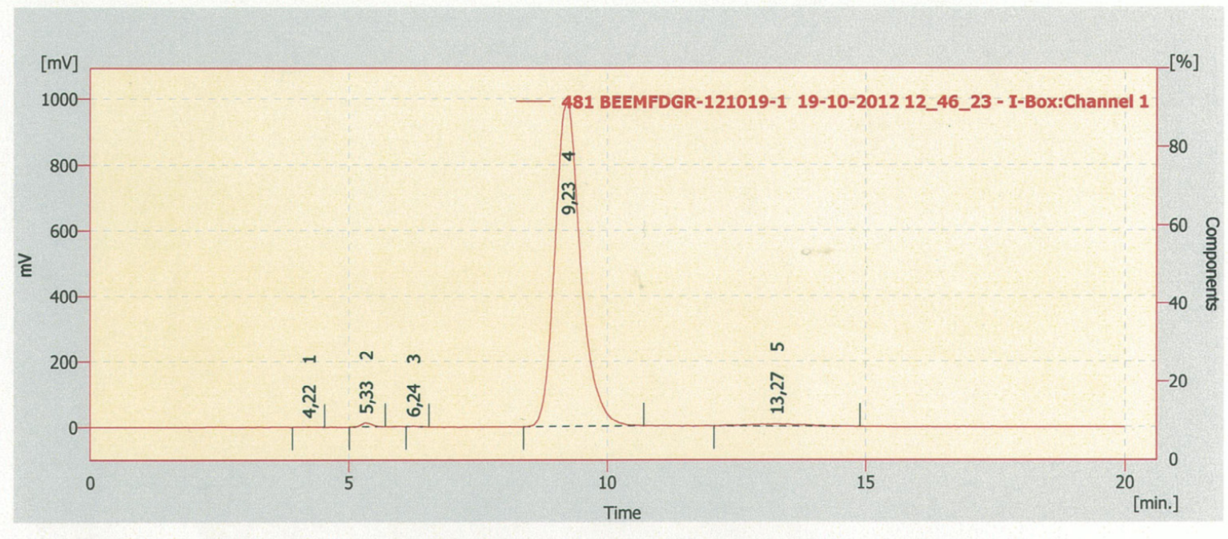

Figure S1 zoom. No [^{18}F]FDM with ret. time 8.2 was detected.

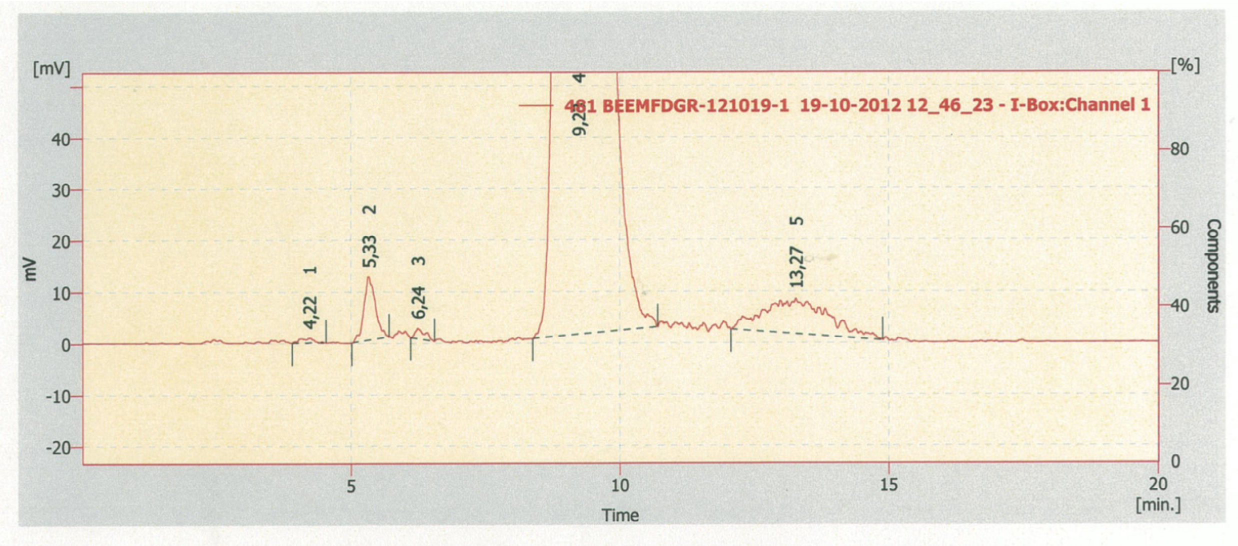

Result Table (Uncal - 481 BEEMFDGR-121019-1 19-10-2012 12_46_23 - I-Box:Channel 1)

	Reten. Time [min]	Area [mV.s]	Height [mV]	Area [%]	Compound Name
1	4,220	17,89105	0,9728968	0,051	
2	5,327	181,97381	12,2411836	0,519	
3	6,240	27,34411	2,0358989	0,078	
4	9,233	34275,01398	990,6622955	97,759	
5	13,273	558,62731	6,8433093	1,593	
	Total	35060,85026	1012,755584	100,000	

Figure S2. Overlay of RI (blue) and radiodetector (red) trace of [^{18}F]FDG – no [^{19}F]FDG detected.

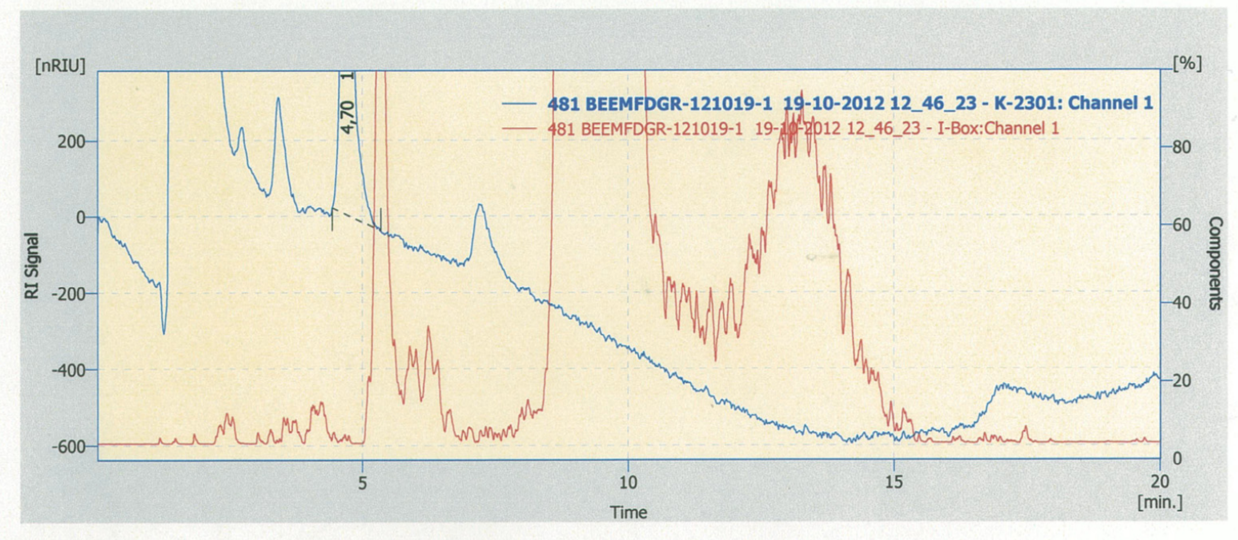

Figure S3. TCL trace of [^{18}F]FDG.

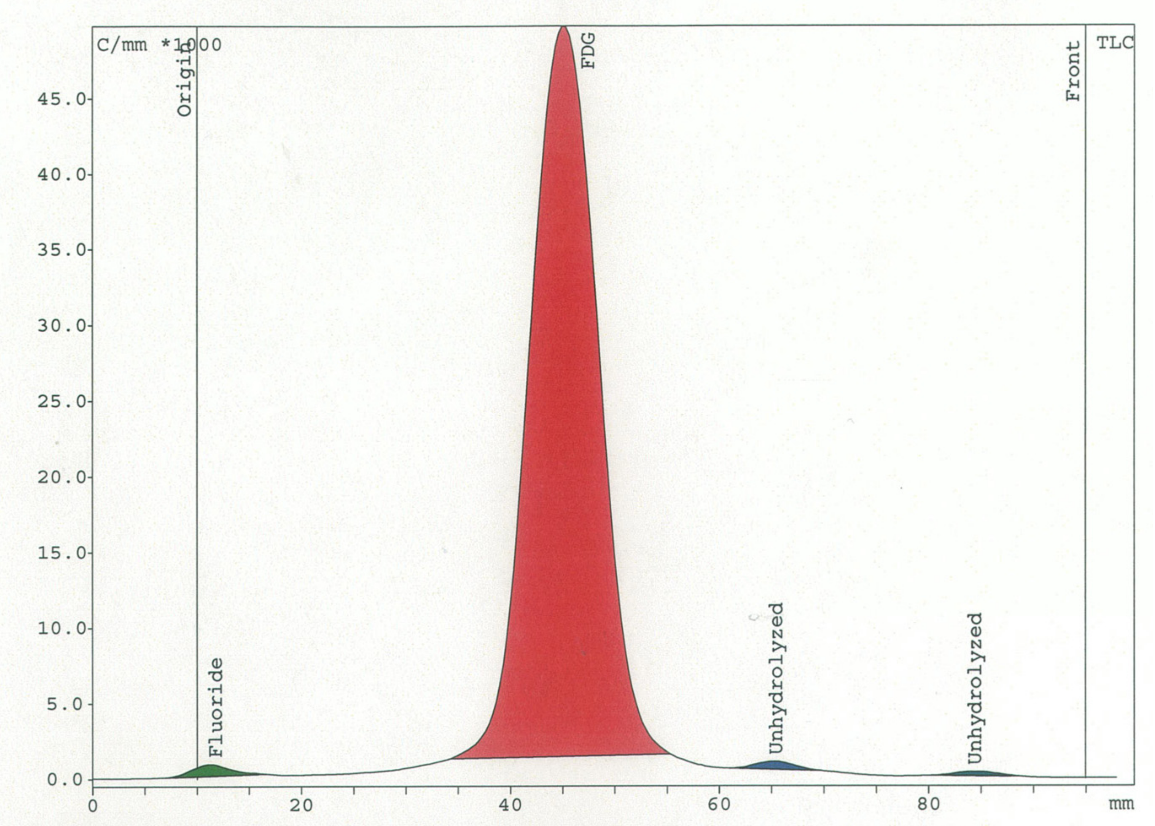

Integration TLC

Substance	R/F	Area Counts	%Area %
Fluoride	0.02	3407.0	0.91
FDG	0.41	366779.0	98.05
Unhydrolyzed	0.65	2398.4	0.64
Unhydrolyzed	0.87	1487.1	0.40
Sum in ROI		374071.6	
Area RF		429642.0	
Ext. BKG		0.00 C/mm	
